# *QuickStats:* Age-Adjusted Rates* of Firearm-Related Homicide,^†^ by Race, Hispanic Origin, and Sex — National Vital Statistics System, United States, 2019

**DOI:** 10.15585/mmwr.mm7042a6

**Published:** 2021-10-22

**Figure:**
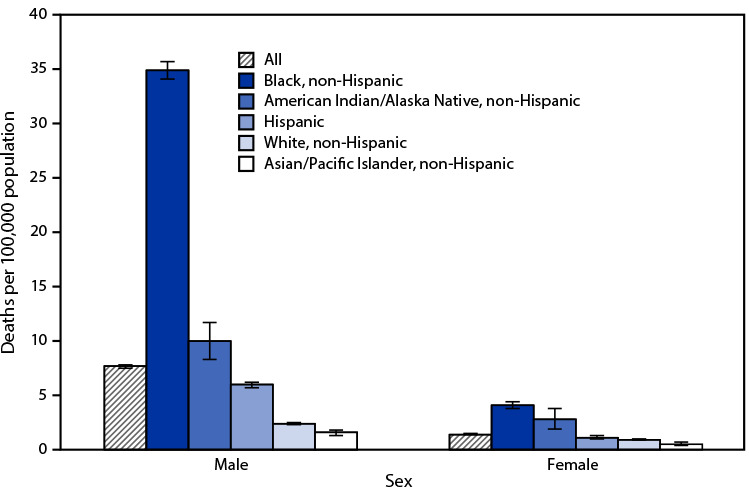
In 2019, among males, non-Hispanic Black males had the highest age-adjusted rate of firearm-related homicide at 34.9 per 100,000 population and non-Hispanic Asian/Pacific Islander males had the lowest rate (1.6). Among females, non-Hispanic Black females had the highest rate (4.1) and non-Hispanic Asian/Pacific Islander females had the lowest rate (0.5). Males had higher rates than females across all race and Hispanic origin groups.

**Source:** National Vital Statistics System, Mortality Data, 2019. https://www.cdc.gov/nchs/nvss/deaths.htm

For more information on these topics, CDC recommends the following link: https://www.cdc.gov/violenceprevention/firearms

